# Detection of 8-Hydroxy-2′-Deoxyguanosine Biomarker with a Screen-Printed Electrode Modified with Graphene

**DOI:** 10.3390/s19194297

**Published:** 2019-10-04

**Authors:** Codruta Varodi, Florina Pogacean, Maria Coros, Marcela-Corina Rosu, Raluca-Ioana Stefan-van Staden, Emese Gal, Lucian-Barbu Tudoran, Stela Pruneanu, Simona Mirel

**Affiliations:** 1 National Institute for Research and Development of Isotopic and Molecular Technologies, 67-103 Donat Street, Cluj-Napoca 400293, Romania; 2 Laboratory of Electrochemistry and PATLAB, National Institute of Research for Electrochemistry and Condensed Matter, 202 Splaiul Independentei Street, Bucharest-6 060021, Romania; 3 Faculty of Applied Chemistry and Material Science, Politehnica University of Bucharest, Bucharest RO-060042, Romania; 4 Department of Chemistry and Chemical Engineering, Hungarian Line of Study, Babes-Bolyai University, 11 Arany János St., Cluj-Napoca 400028, Romania; 5 Faculty of Biology and Geology, Electron Microscopy Lab, Babes-Bolyai University, 5-7 Clinicilor St., Cluj-Napoca RO-400015, Romania; 6 Faculty of Pharmacy, “Iuliu Hatieganu” University of Medicine and Pharmacy, Cluj-Napoca 400349, Romania

**Keywords:** 8-hydroxy-2′-deoxyguanosine, graphene, electrochemical detection, screen-printed electrodes

## Abstract

In this work we present the preparation of graphene material by exfoliation of graphite rods via pulses of current in electrolyte, containing a mixture of boric acid (0.05 M) and sodium chloride (0.05 M). The material was morphologically and structurally characterized by SEM/TEM/HR-TEM, XRD and FTIR techniques. TEM investigation of graphene flakes deposited onto carbon-coated grids allowed the visualization of thin and transparent regions, attributed to few-layer graphene (FLG), as well as thick and dark regions attributed to multi-layer graphene (MLG). The mixed composition of the material was additionally confirmed by XRD, which further indicated that the amount of FLG within the sample was around 83%, while MLG was around 17%. The performance of a screen-printed electrode (SPE) modified with graphene (SPE-Gr) was tested for 8-hydroxy-2′-deoxyguanosine detection. The graphene-modified electrode had a higher sensitivity in comparison with that of SPE, both in standard laboratory solutions (phosphate buffered saline—PBS) and in human saliva.

## 1. Introduction

8-hydroxy-2′-deoxyguanosine (8-OHdG) is one of the markers used for the evaluation of oxidative damage in DNA, and is a risk factor for a variety of diseases [1,2]. Accumulation of 8-OHdG has been reported in cancerous tissues [3,4], atherosclerosis and diabetes [5], polycystic ovary syndrome, fertility and sterility [6] leukemia [7], or in chronic and aggressive periodontitis [8,9,10]. Several techniques were used for the analysis of biological samples such as high-performance capillary electrophoresis [11], gas chromatography-mass spectrometry [12], HPLC with electrochemical detection [13], post labeling methods [14] and electrochemical methods [15]. All of the above-mentioned detection techniques require sophisticated and laborious technologies with high costs and long response time. By comparison, electrochemical methods are more convenient, easy to operate and cost-effective. Being amongst the most promising materials for electrochemical sensors fabrication, graphene proved to be an effective sensing material for pursuing experiments in the selective detection of biomolecules [16,17]. 

Amongst the new ways of graphene preparation [18], graphite exfoliation by electrochemical methods [19] has drawn increasing attention in recent years. Compared with the conventional synthesis methods, the electrochemical exfoliation of graphite has the advantage of lower cost, shorter reaction time, easy operation, and being more suited to mass production of graphene [20]. Usually, the exfoliation takes place at room temperature in mild electrolytes, without strong oxidants and heat-oxidation effects, and is considered an environmentally friendly method for the graphene synthesis. The obtained graphene has high crystallinity and low oxidation degree, thus being very convenient as material for electrodes modification [21]. 

Liu et al. [22] reported the modification of a glassy carbon electrode with poly(l-arginine) and graphene-wrapped Au nanoparticles, used for the electrochemical detection of 8-OHdG. The modified electrode was also tested in human urine samples with good recovery values. By combining single-stranded DNA with graphene nanosheets, a novel biosensor with high electrocatalytic activity towards 8-OHdG oxidation was fabricated [23]. In our previous work [24], two graphene-based materials, synthesized by a new exfoliation method using short electrical current pulses, were used to modify glassy carbon electrodes for the subsequent detection of 8-OHdG. Recently, stochastic sensors were used to develop a pattern recognition method of 8-OHdG using graphene decorated with different types of nanoparticles (TiO_2_Ag or TiO_2_Au) [25]. The sensors exhibited high sensitivities and low determination limits for 8-OHdG, which may be beneficial for early detection of leukemia.

Screen-printed electrodes (SPE) offer some advantages over traditional electrodes (glassy carbon; gold) as an economical transducer with small size, large mass production and the possibility to be attached to a portable device which facilitates in-situ applications [26,27,28]. In addition, the carbon inks used to imprint the working electrode can be widely modified by the addition of materials and/or molecules and this versatility confers the capacity to be used in biomedical analyses [29]. A typical screen-printed electrode contains the counter electrode on the same substrate the working electrode where the electrochemical reaction occurs, which allows the current to flow to the working electrode and the reference electrode, which provides the reference value of the potential. 

The purpose of the present study was to evidence the performances of screen-printed electrodes modified with graphene towards the detection of 8-OHdG. SPE are normally designed as single-use electrodes; therefore, they can be employed in clinical analyses. Hence, we tested the electrochemical performance of a screen-printed electrode modified with graphene towards 8-OHdG detection, both in standard laboratory solutions (pH6 PBS) and in human saliva, and compared the results with those of unmodified SPE. To the best of our knowledge, there are only a few studies [30,31] that show the performance of SPE and SPE-Gr electrodes towards the detection of 8-OHdG; therefore, our experimental results are valuable. 

## 2. Materials and Methods

### 2.1. Chemicals

8-hydroxy-2′-deoxyguanosine (8-OHdG) was purchased from Cayman Chemical Company. Boric acid (H_3_BO_3_), sodium chloride (NaCl), monobasic sodium phosphate (NaH_2_PO_4_), and dibasic sodium phosphate (Na_2_HPO_4_) were bought from REACTIVUL Bucuresti (Bucharest, Romania). Potassium ferrocyanide K_4_[Fe(CN)_6_] and KCl were purchased from Merck. Dimethylformamide (DMF) was purchased from JTBaker (HPLC grade). High-purity (99.9995%) graphite rods were employed for electrochemical exfoliation and were bought from Alfa-Aesar (Kandel, Germany). 

### 2.2. Instruments

For SEM/TEM analysis, we employed a Hitachi SU8230 (Japan) High-Resolution Scanning Transmission Electron Microscope (200 kV accelerating voltage). 

For HRTEM imaging, a TecnaiG2 F20 XTWIN electron microscope was used (200 kV accelerating voltage). The sample, previously dispersed in ethanol, was dropped onto a lacey carbon/formvar film cooper grid and allowed to dry at room temperature for few minutes. 

The X-ray powder diffraction (XRD) patterns were collected with DIFFRAC plus XRD Commander, at a scanning speed of 0.020 s-1 with a Bruker D8-Advance Diffractometer (Germany) (40 kV and 40 mA) using CuKα1 radiation (λ = 1.5406 Å). The structural parameters were obtained after the diffraction patterns were background corrected. 

For Fourier-Transformed Infrared (FTIR) measurements, we employed a Bruker Tensor II spectrometer (Germany). 

A Potentiostat/Galvanostat Instrument (PGSTAT-302N, Metrohm-Autolab B.V., Netherlands) connected to a standard cell (three-electrodes), was used for the electrochemical measurements (Cyclic Voltammetry—CV; Linear Sweep Voltammetry—LSV and Electrochemical Impedance Spectroscopy—EIS). 

### 2.3. Graphene Synthesis by Pulse Exfoliation of Graphite Rods

**Figure 1:**
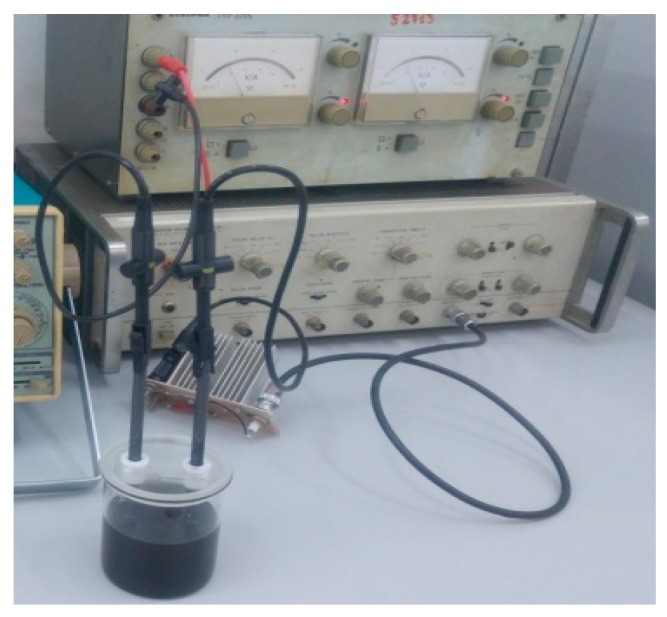
Experimental set-up employed for graphite electrochemical exfoliation by pulses of current (0.5 A intensity; 2.5 s pulse duration; 0.8 s pause between two pulses).

The graphene synthesis by pulses of current was developed in our group as previously reported [24]. By applying short electrical current pulses (0.5 A intensity; 2.5 s pulse duration; 0.8 s pause) between two graphite bars immersed in the appropriate electrolyte (0.05 M boric acid + 0.05 M NaCl), exfoliation of the bars was induced (Figure 1). After 7 h, the process was terminated and the material was left in the solution overnight. The next day, it was washed with a large amount of water, then sonicated for 2 h, filtered and dried by liophylization. The ionic species involved in the electrochemical exfoliation of graphite rods were H^+^, Na^+^ (at cathode) and BO_3_^3−^ and Cl^−^ (at anode). We have to mention that the exfoliation was extremely slow when the electrolyte contained NaCl without boric acid. Only a few milligrams of material were collected after several hours, so the presence of bulky ions like BO_3_^3−^ proved essential for graphite exfoliation.

### 2.4. Modification of Screen-Printing Electrode with Graphene (SPE-Gr)

**Scheme 1:**
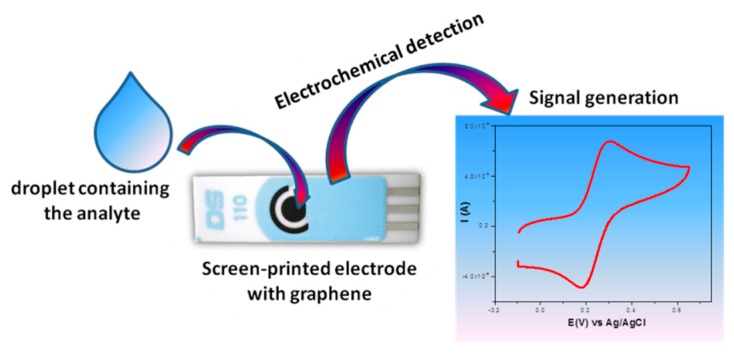
Representation of a screen-printed electrode modified with graphene and its application for the detection of 8-hydroxy-2′-deoxyguanosine.

The screen-printed electrode (SPE) modified with graphene (SPE-Gr) was fabricated by drop-casting on its surface a certain volume (9.5 μL) of graphene, which was previously dispersed in DMF (1 mg/mL concentration) (Scheme 1).

## 3. Results and Discussions

### 3.1. Morphological and Structural Characterization of Graphene

**Figure 2:**
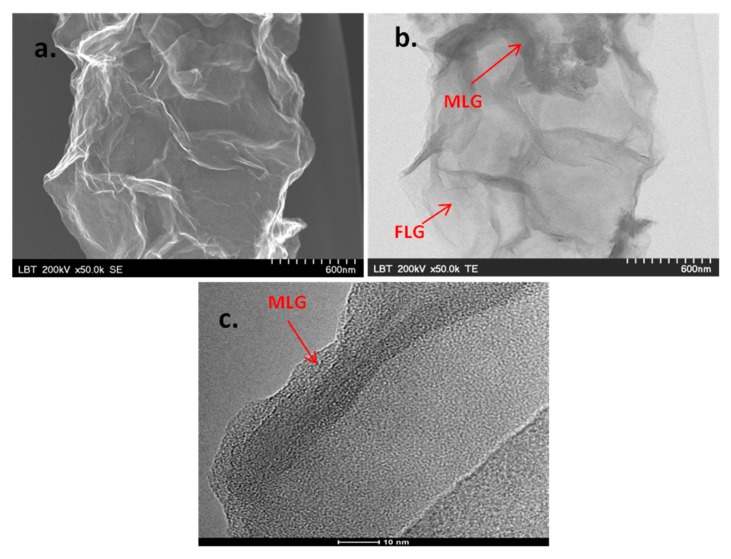
SEM image of the graphene sample—scale bar 600 nm (**a**); TEM image of the sample, revealing few- and multi-layer graphene—scale bar 600 nm (**b**); HR-TEM image of the edge of multi-layer graphene—scale bar 10 nm (**c**).

The morphological characteristics of the graphene sample were investigated by SEM/TEM/HR-TEM techniques. As can be seen in Figure 2a (SEM image), the sample consists of large sheets, with a length of hundreds of nanometers. The white lines correspond to regions with wrinkles, folding and overlapping of the sheets. The TEM image, presented in Figure 2b, reveals graphene flakes formed by thin and transparent regions, attributed to few-layer graphene (FLG) and dark regions attributed to multi-layer graphene (MLG). MLG is generally formed due to strong π–π stacking interactions between several single-layer graphene sheets. A representative HR-TEM image of the edge of multi-layer graphene can be seen in Figure 2c. 

**Figure 3:**
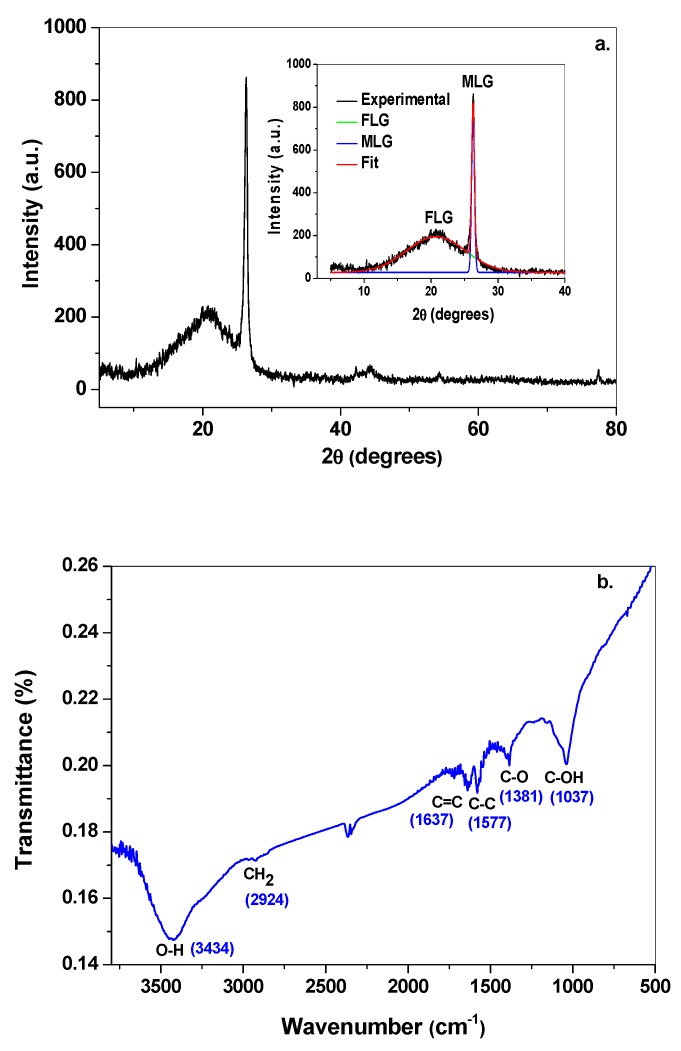
The XRD pattern of the graphene sample: recorded (black line); inset: deconvolution of the spectrum in FLG (green line), MLG (blue line), fitted spectrum (red line) (**a**); FTIR spectrum of graphene (**b**).

Next, from the X-ray Powder Diffraction pattern of the graphene sample, we determined some important structural parameters: the inter-layer distance (*d*), the mean crystallites size (*D*), and the number (*n*) of graphene layers within a flake. The amount (%) of few- and multi-layer graphene sheets present in the sample was calculated by dividing the area of each peak by the total area of the pattern. In Table 1 and Figure 3a, it is shown that the synthesized sample contains 83% FLG and 17% MLG. The *d* distance is relatively large in FLG (0.475 nm) and smaller in MLG (0.376 nm). Both distances are larger than that of graphite (0.335 nm) due to the presence of some oxygen-containing groups attached to graphene layers, as proven by FTIR investigation (Figure 3b).

The FTIR spectrum of graphene allows the identification of several characteristic absorption bands, in good agreement with the literature [32]. The adsorbed water in the sample is shown by the broad peak from 3000 to 3600 cm^−1^ representing the hydroxyl (O–H) stretching vibrations, and by the peak at 1037 cm^−1^ attributed to C–OH in plane vibration. The CH2 stretching vibration (2924 cm^−1^), the sp2-hybridized C=C in-plane vibration (1637 cm^−1^), C–C stretching (1577 cm^−1^), as well as C–O in-plane vibration at 1381 cm^−1^ are also present. The peak from 1381 cm^−1^ may appear due to the partially oxygenated groups attached to the graphene surface. Their presence in a small amount is supported by the absence of GO peak in the XRD pattern of the sample.

### 3.2. Morphological Characterization of the Substrate

**Figure 4:**
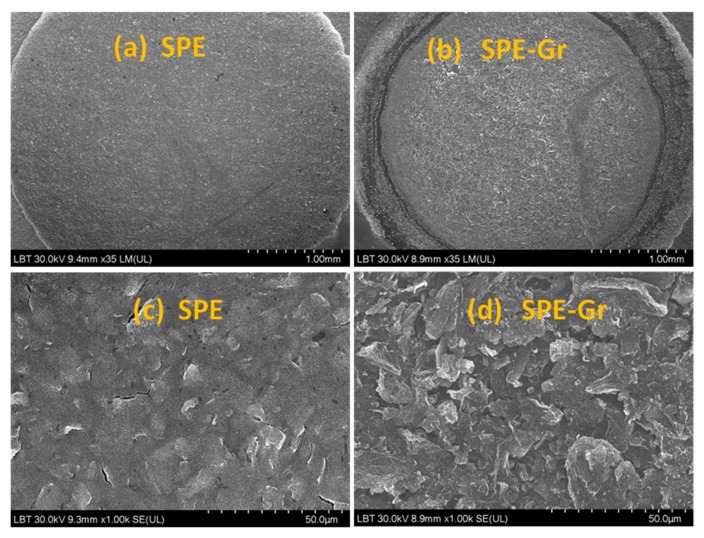
Low- and high-magnification SEM images of SPE (**a**,**c**) and SPE-Gr (**b**,**d**); scale bar: 1 mm (**a**,**b**); 50 µm (**c**,**d**).

Prior to testing the electrocatalytic performances of SPE and SPE-Gr electrodes towards the 8-OHdG detection, a scanning electron microscopy investigation was carried out. Hence, SPE was examined before and after modification with graphene flakes, as can be seen in Figure 4. In the case of SPE (Figure 4a,c: low and high magnification, respectively), it can be seen that the morphology is relatively flat. As expected, after the addition of graphene (Figure 4b,d: low and high magnification, respectively), the surface roughness has changed. Large graphene platelets can be observed on top of the graphite substrate with beneficial effects towards the detection of 8-OHdG. 

### 3.3. Electrochemical Studies 

**Figure 5:**
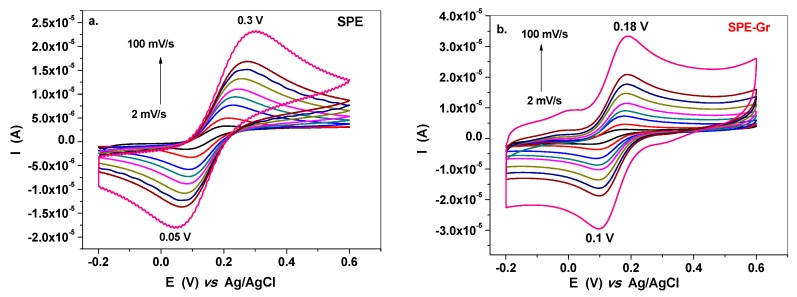

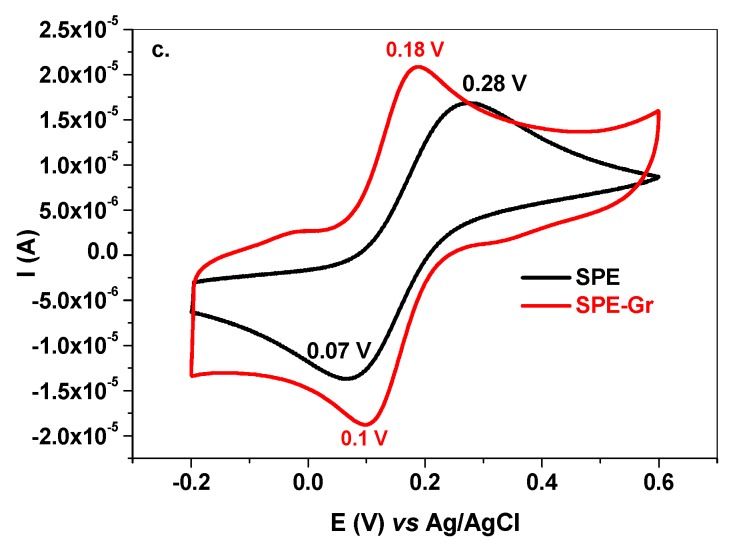
CVs recorded with SPE (**a**) and SPE-Gr (**b**) in the presence of 10^−3^ M K_4_[Fe(CN)_6_] in 0.2 M KCl supporting electrolyte at various scanning rates, from 2 to 100 mV/s; comparison between two CVs recorded with SPE and SPE-Gr; 50 mV/s scan rate (**c**).

The first CV experiments were performed in the presence of K4[Fe(CN)6] (1 × 10^−3^ M + 0.2 M KCl; scan speed 2–100 mV/s (Figure 5). In the case of SPE (Figure 5a), the peak potential separation was very large (~ 250 mV), which is characteristic of a quasi-reversible process. For SPE-Gr, the electrochemical signal was higher and the peak potential separation was smaller (80 mV), being close to the value characteristic for a reversible process (Figure 5b). The formal potential (E0′) was also different for the two electrodes (0.175 V for SPE and 0.14 V for SPE-Gr, at 50 mV/s), proving that the oxidation of K_4_[Fe(CN)_6_] was highly favored in the case of the graphene-modified electrode. The comparison between two CVs (50 mV/s scan rate) recorded with SPE and SPE-Gr can be seen in Figure 5c. 

**Figure 6:**
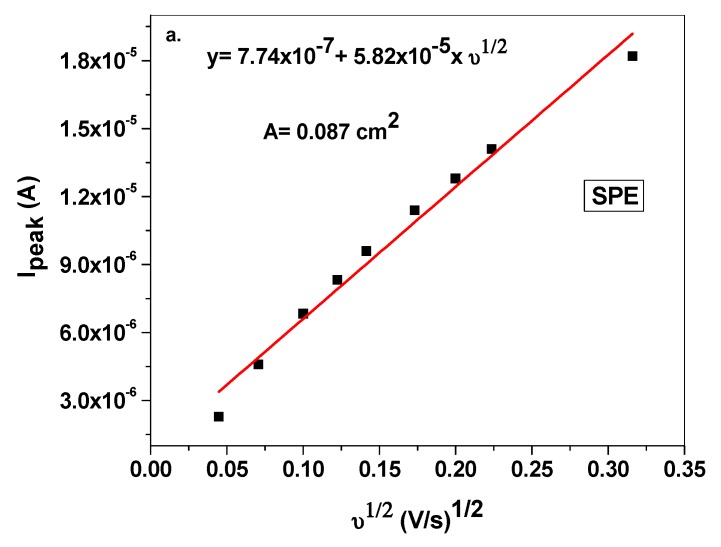

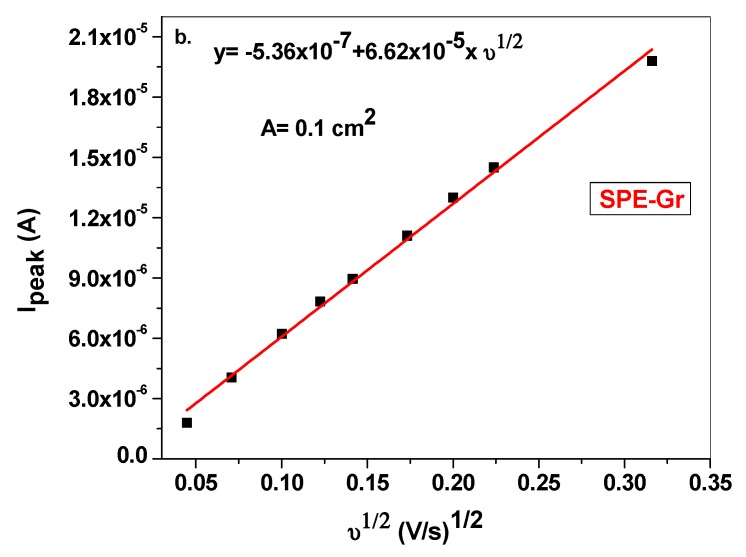
The linear plot showing the variation of the anodic peak current (*I*_peak_) vs. *ʋ*^1/2^ for (**a**) SPE and (**b**) SPE-Gr.

In order to determine the active areas of each electrode, we employed the (Ipeak) vs. *ʋ*^1/2^ plot (Figure 6), and also the Randles–Sevcik Equation (1) [33]:

*I*_peak_ = ±2.687 × 10^5^*AD*^1/2^*n*^3/2^*Cʋ*^1/2^ (1)

where *I*_peak_—the intensity of the anodic peak (*A*); *D*—diffusion coefficient of K_4_[Fe(CN)_6_] (6.2 × 10^−6^ cm^2^/s); *A*—the active area (cm^2^) of the bare or modified electrode; *n*—the number of electrons transferred during the oxidation/reduction process; *C*—the concentration of [K_4_Fe(CN)_6_] in solution (mol/cm^3^) and *ʋ*—the scan rate (V/s). 

From each slope (Figure 6), the values of the active areas of SPE and SPE-Gr were determined to be 0.087 and 0.1 cm^2^, respectively. Although the active area of the modified electrode was not much larger than that of the bare electrode, the electrochemical signal obtained for the detection of 8-OHdG was considerably improved. This may be related with the heterogeneous electron transfer rate constant, which was determined from the impedance spectra. The EIS technique provides a better description of the solution/electrode interface than CV does; therefore, it was used to investigate the electron transfer kinetics. The EIS measurements were recorded at the formal potential of each electrode, previously determined from CV (E0′ = 0.175 V for SPE and 0.14 V for SPE-Gr).

**Figure 7:**
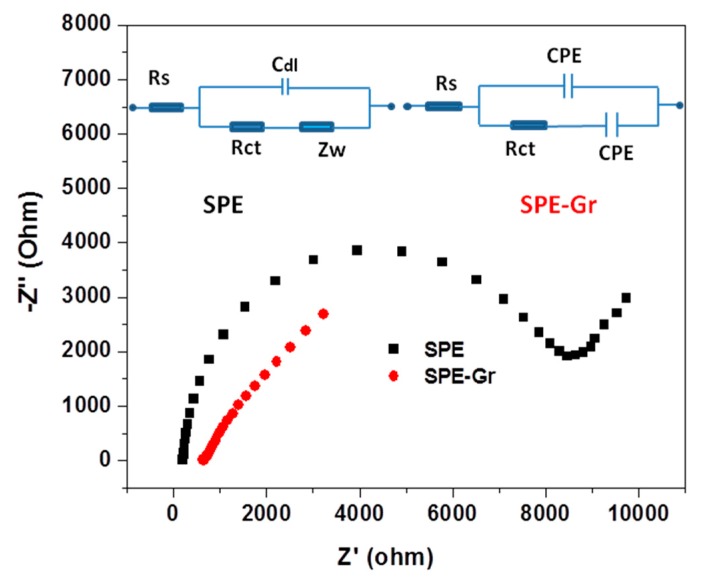
EIS spectra of SPE (black) and SPE-Gr (red) recorded in 1 × 10^−3^ M K_4_[Fe(CN)_6_] solution (0.2 M KCl supporting electrolyte); Inset: equivalent electrical circuits of the electrochemical interface used to fit the impedance spectrum of SPE and SPE-Gr: *R*_s_—the solution resistance; *Z*_w_—the Warburg impedance; *R*_ct_—the charge-transfer resistance; *C*_dl_—double-layer capacitance; CPE—Constant Phase Element.

The recorded EIS spectrum for SPE can be seen in Figure 7 (black) and it was interpreted based on an equivalent electrical circuit (inset) that contains the solution resistance (*R*_s_), the Warburg impedance (*Z*_W_) due to the diffusion of ions in solution, the charge-transfer resistance (*R*_ct_) and the double-layer capacitance (*C*_dl_) [34]. In the case of SPE-Gr (Figure 7—red), it was not possible to model the data using only finite diffusion; therefore, *C*_dl_ and *Z*_W_ components were replaced by Constant Phase Elements (CPE). CPE arose due to the roughness of the electrode surface and dynamic disorder associated with the diffusion [35]. 

The EIS experimental data were fitted with the proposed equivalent electrical circuits in order to find the *R*_ct_ values for bare and graphene-modified electrode. Hence, the bare SPE had a very large *R*_ct_ value (7240 Ω), indicating a sluggish transfer of electrons at the solution/electrode interface. SPE-Gr had a considerably lower value for *R*_ct_ (375 Ω), which may be favourable for the electron transfer during the redox process.

In order to determine the apparent heterogeneous electron transfer rate constant (*K*_app_) for each electrode, we employed the following equation [36]:

(2) $$K_{\mathrm{app}}=\frac{RT}{n^{2}F^{2}AR_{\mathrm{ct}}C}$$

where *R* is the ideal gas constant (8.314 Joule/(mol·K)); T is the temperature (298 K); F is the Faraday constant (96485 C/mol); n is the number of electrons transferred during the redox reaction (*n* = 1); *A* is the active area of the electrode (cm^2^); *R*_ct_ is the charge-transfer resistance obtained from the fitted Nyquist plots (Ω); *C* is the concentration of the redox specie (mol/cm^3^). 

Kapp is influenced by many factors such as the roughness, the surface functional groups and the edge plane defects on the surface [37]; therefore, we expected to see differences between the values of SPE and SPE-Gr. Indeed, in the case of bare SPE, *K*_app_ was determined to be 4.15 × 10^−4^ cm/s, being considerably smaller than that corresponding to SPE-Gr (6.9 × 10^−3^ cm/s). This proves that the attached graphene layer not only increases the active area but also highly promotes the transfer of electrons from the redox specie to the electrode surface. 

According to Banks et al., [38] graphene has a large basal plane and low edge plane content; therefore, it exhibits slow electron transfer kinetics. Their DFT calculations show that there is a higher electron density around the edge of the graphene in comparison to the basal plane. In our case, the sample contained not only few-layer but also multi-layer graphene (17%), which brought a high proportion of edge planes and, consequently, increases the value of Kapp with beneficial effects towards the detection of 8-OHdG.

**Figure 8:**
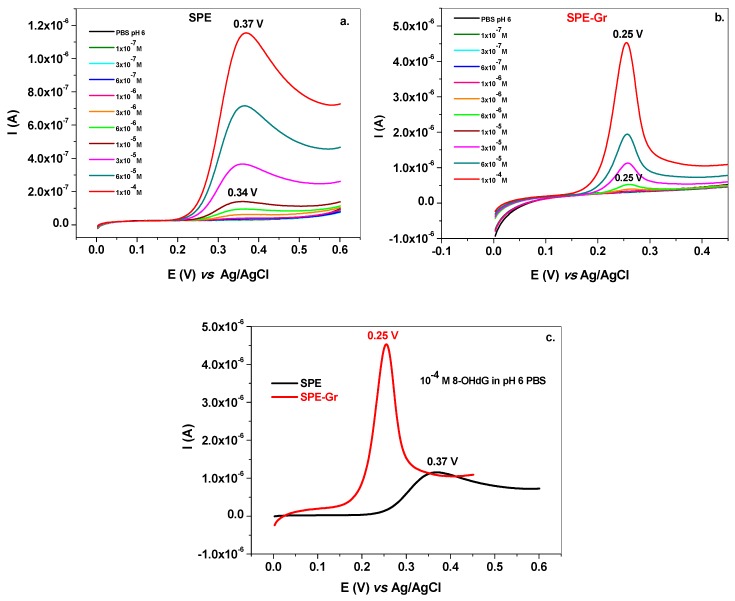
LSVs recorded with SPE (**a**) and SPE-Gr (**b**) in pH 6 PBS solutions containing various concentrations of 8-OHdG (1 × 10^−7^–1 × 10^−4^ M); comparison between the SPE and SPE-Gr signals at one selected 8-OHdG concentration (1 × 10^−4^ M in pH 6 PBS) (**c**); 10 mV/s scan rate.

This can be seen in Figure 8a–c, where the electrochemical signals of SPE and SPE-Gr in the presence of 8-OHdG are shown. The LSVs were recorded in standard laboratory solutions (pH6 PBS) containing increasing concentrations of 8-OHdG (from 1 × 10^−7^ to 1 × 10^−4^ M). In the case of SPE, the current signal was relatively broad and the value of the peak potential is influenced by the 8-OHdG concentration, varying from +0.34 to +0.37 V. In contrast, for the SPE-Gr, the current signal was five times larger than that of SPE and the 8-OHdG concentration had no influence on the peak potential (+0.25 V). A clear comparison between the SPE and SPE-Gr signals at one selected 8-OHdG concentration (1 × 10^−4^ M in pH 6 PBS) can be seen in Figure 8c (10 mV/s scan rate).

**Figure 9:**
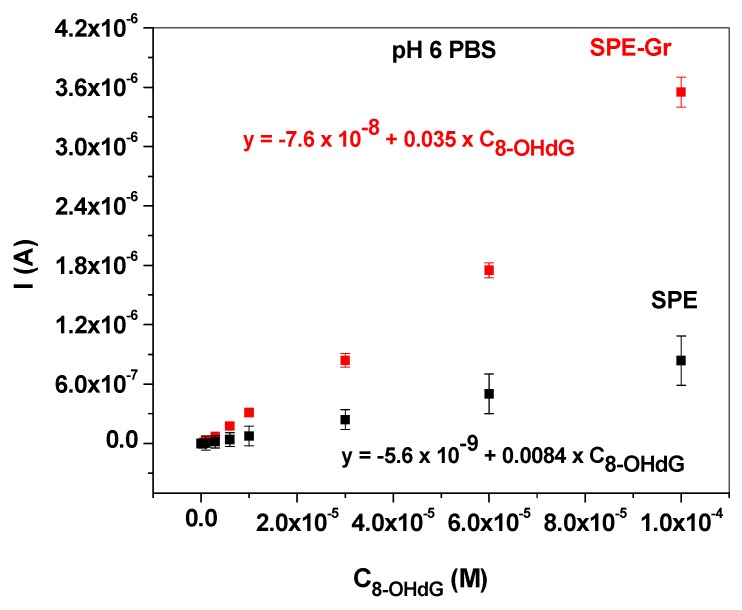
Calibration plot of SPE (black) and SPE-Gr (red) obtained in pH 6 PBS solutions containing various concentrations of 8-OHdG.

By representing the oxidation peak signal (Ipeak) vs. 8-OHdG concentration, the corresponding calibration plot for each electrode was obtained (Figure 9). The sensitivity of SPE-Gr (0.035 A/M) was four times higher than that of SPE (0.0084 A/M). In addition, it had a wider linear range (3 × 10^−7^–1 ×10^−4^ M in comparison with 6 × 10^−7^–1 × 10^−4^ M) and lower limit of detection (LOD = 9 × 10^−8^ M in comparison with 1.8 × 10^−7^ M; S/N = 3.3).

Although the screen-printed electrodes are normally designed for single-use, we evaluated the time stability of SPE and SPE-Gr by performing calibration measurements on different days. After each measurement, the electrodes were thoroughly washed with distilled water and then kept dry at room temperature. It became clear that the reproducibility was relatively poor in the case of SPE electrode (RSD 11.5%) possible due to the adsorption of the oxidation products on its surface. In comparison, SPE-Gr had an excellent reproducibility (RSD < 3%). The results of several repetitive experiments confirmed that the graphene material had an excellent adhesion to the electrode surface and consequently, the modified electrode had good reproducibility and stability.

A comparison of SPE-Gr performances with those of other types of modified electrodes can be seen in Table 2.

In order to investigate the applicability of SPE and SPE-Gr for the assessment of 8-OHdG level in real samples, we employed human saliva as electrolyte. Samples of saliva were collected from healthy patients and diluted with pH6 PBS (1:4), then centrifuged for five minutes (8000 rpm). The supernatant was removed and then used to prepare solutions with various concentrations of 8-OHdG (6 × 10^−6^–6 × 10^−4^ M). We have to emphasize that the concentration range in saliva sample is different than that used in standard pH 6 PBS solution. The reason for this relates to the fact that below 6 × 10^−6^ M 8-OHdG, no reliable signal was recorded with SPE-Gr. In the case of SPE, no signal was recorded below 10^−5^ M, therefore, the chosen concentration range was between 1 × 10^−5^–6 × 10^−4^ M. 

**Figure 10:**
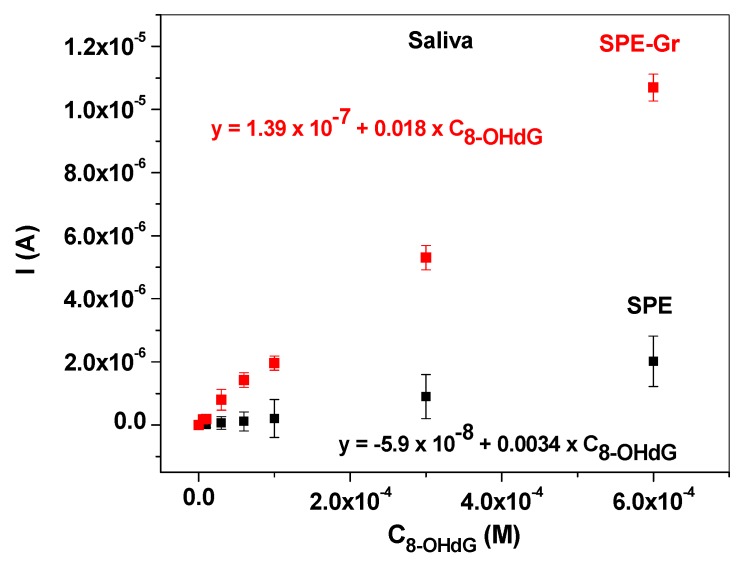
Calibration plots for SPE-Gr (red) and SPE (black) obtained in saliva solutions.

In Figure 10 the 8-OHdG calibration plots obtained with SPE and SPE-Gr in diluted saliva solutions are presented. Similarly, with the calibration plots in standard pH6 PBS, SPE-Gr had a higher sensitivity (0.018 A/M) in comparison with that of SPE (0.0034 A/M) and a better reproducibility. In addition, the limit of detection (LOD) was lower (1.8 × 10^−6^ M) in comparison with that of SPE (3 × 10^−6^ M). However, due to the complex composition of saliva, the linear range was narrow for both electrodes: 6 × 10^−6^–6 × 10^−4^ M for SPE-Gr and 1 × 10^−5^–6 × 10^−4^ M for SPE. 

Finally, known concentrations of 8-OHdG in a spiked saliva sample were determined using SPE-Gr. As can be seen in Table 3, good recoveries of 8-OHdG concentration were obtained, proving the applicability of graphene-modified electrode in real sample analysis.

## 4. Conclusions

In the present work, graphene (Gr) was synthesized by exfoliation of graphite rods with pulses of current (0.5 A intensity; 2.5 s pulse duration; 0.8 s pause between two pulses) in the appropriate electrolyte (0.05 M boric acid + 0.05 M NaCl). After the material was morphologically and structurally characterized with advanced techniques (SEM/TEM/HR-TEM, XRD, and FTIR), it was deposited by drop-casting onto the surface of a screen-printed electrode (SPE-Gr). The electroactive area and the heterogeneous electron transfer rate constant (Kapp) of bare and graphene-modified SPE were determined and compared. Although the active area of SPE-Gr (0.1 cm^2^) was not much larger than that of SPE (0.087 cm^2^), the electrochemical signal obtained for the detection of 8-OHdG was considerably higher. We suggest that the amount of multi-layer graphene (17%) present in the sample results in a high proportion of edge planes and consequently, increases the value of the heterogeneous electron transfer rate for SPE-Gr, with beneficial effects towards the detection of 8-OHdG.

## Figures and Tables

**Table 1:** Structural parameters obtained from the XRD pattern.

Sample	*2θ* (°)	*D* (nm)	*d* (nm)	*n*	%
Gr	20.7 (FLG)	0.96	0.475	∼2	83
	26.3 (MLG)	22.19	0.376	~58	17

**Table 2:** Comparison of SPE-Gr performances with those of other types of modified electrodes.

Electrode/Sensing Material	Linear Range (In Standard PBS Solutions)	LOD (nM)	Ref.
GC/carbon nanotubes dispersed in polyethylenimine	5 × 10^−7^ M–3 × 10^−5^ M	100	[15]
SPCE/multi-layer-based graphene oxide covered zinc oxide nanoflower	0.05–536.5 μM	36.6	[30]
SPCE/dysprosium oxide nanoparticles@rGO	0.05–315.3 μM	1.02	[31]
GC/electrochemically reduced graphene oxide/multiwalled carbon nanotubes	3–75 µM	35	[39]
GC/Graphene/Nafion	0.07–33.04 μM	1.12	[40]
GC/MWCNTs	6.08 × 10^−6^−1.64 × 10^−5^ M	18.8	[41]
SPE-Gr	3 × 10^−7^–1 × 10^−4^ M	90	This work

**Table 3:** Determination of 8-OHdG with SPE-Gr in spiked saliva samples.

Electrode	Added (M)	Found (M)	Recovery (%)
SPE-Gr	1 × 10^−5^	0.95 × 10^−5^	95
	6 × 10^−5^	6.38 × 10^−5^	106
